# A review on the neural bases of episodic odor memory: from laboratory-based to autobiographical approaches

**DOI:** 10.3389/fnbeh.2014.00240

**Published:** 2014-07-07

**Authors:** Anne-Lise Saive, Jean-Pierre Royet, Jane Plailly

**Affiliations:** Olfaction: from Coding to Memory Team, Lyon Neuroscience Research Center, CNRS UMR 5292—INSERM U1028—University Lyon1Lyon, France

**Keywords:** episodic memory, recognition memory, autobiographical memory, olfaction, behavior, approaches, neural bases, human

## Abstract

Odors are powerful cues that trigger episodic memories. However, in light of the amount of behavioral data describing the characteristics of episodic odor memory, the paucity of information available on the neural substrates of this function is startling. Furthermore, the diversity of experimental paradigms complicates the identification of a generic episodic odor memory network. We conduct a systematic review of the literature depicting the current state of the neural correlates of episodic odor memory in healthy humans by placing a focus on the experimental approaches. Functional neuroimaging data are introduced by a brief characterization of the memory processes investigated. We present and discuss laboratory-based approaches, such as odor recognition and odor associative memory, and autobiographical approaches, such as the evaluation of odor familiarity and odor-evoked autobiographical memory. We then suggest the development of new laboratory-ecological approaches allowing for the controlled encoding and retrieval of specific multidimensional events that could open up new prospects for the comprehension of episodic odor memory and its neural underpinnings. While large conceptual differences distinguish experimental approaches, the overview of the functional neuroimaging findings suggests relatively stable neural correlates of episodic odor memory.

## Introduction

Human episodic memory is the long-term memory process that enables one to mentally and consciously relive specific, personal events from the past (Tulving, 1972, 1983). It is associated with a feeling of mental time travel, a sense of self, and the autonoetic consciousness that allows one to be aware of the subjective time at which events happened (Tulving, 2001, 2002). Although this definition is accepted, episodic memory is experimentally studied through a large set of paradigms that differ in all dimensions of the memory. The content of the memory and the procedures for encoding and retrieval vary in complexity and ecological validity, while the retention time varies in delay. As a consequence, “episodic memory” refers to an ensemble of memory processes. To provide a general picture of episodic memory, it is thus of interest to orient this investigation by the experimental approach. Two different approaches are usually employed to investigate the explicit retrieval of past events: ***laboratory-based approaches*** and ***autobiographical approaches*** (McDermott et al., 2009). In the first case, experimenters test the memorization of artificial episodes created in the laboratory, whereas in the second case, experimenters test the retrieval of real-life memories encoded in the participants’ past. McDermott et al. (2009) further emphasized that the two methods differ in time “*not only in that the events of interest have occurred on different timescales (weeks or years for studies in the autobiographical memory tradition compared with minutes/hours in the laboratory memory tradition): It can take people on the order of 8–12 s to construct a vivid autobiographical memory (Robinson, 1976), compared to recognition memory decisions, which often occur in a second or two*”.

Episodic memory depends on the medial temporal lobe, which is composed of different interconnected subregions, including the hippocampus and adjacent parahippocampal, perirhinal and entorhinal cortices (Milner et al., 1968; Squire, 1992; Cohen and Eichenbaum, 1993). The contribution of each of the medial temporal lobe components to the memory process and their connectivity with the neocortex has been widely investigated (Suzuki and Amaral, 1994; Burwell and Amaral, 1998; Witter et al., 2000; Squire et al., 2004; Davachi, 2006; Diana et al., 2007; Eichenbaum et al., 2007). In summary, the cortical projections encompass two parallel pathways. In one pathway, sensory areas project inputs that are critically involved in object perception onto the perirhinal cortex and hence onto the lateral entorhinal cortex. In the other pathway, the parahippocampal cortex and then the medial entorhinal cortex receive visuospatial information. Both entorhinal cortices then converge onto the hippocampus and allow for the representation of the object in the visuospatial context in which it was experienced.

**Figure 1:**
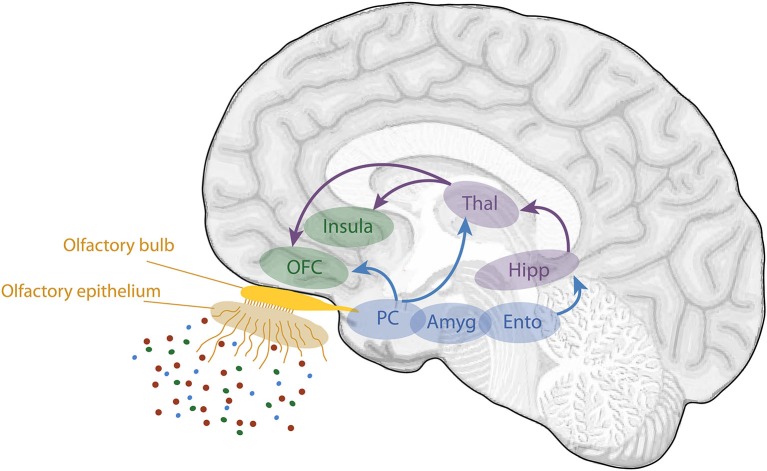
**Schematic view of the human olfactory system**. The primary and secondary olfactory cortices are represented in blue and green, respectively. Amyg, amygdala; Ento, entorhinal cortex; Hipp, hippocampus; OFC, orbitofrontal cortex; PC, piriform cortex; Thal, thalamus (adapted from Royet et al., 2014).

Phenomenologically, the sense of smell demonstrates a close relationship with episodic memory. Odors are well known to be particularly powerful memory cues. Among all sensorial stimuli, odors appear to trigger the most vivid and emotional memories (e.g., Hinton and Henley, 1993; Chu and Downes, 2002; Herz and Schooler, 2002; Larsson and Willander, 2009). This property is usually explained from an anatomical point of view. The olfactory input has direct connections via the olfactory bulb and the primary olfactory (piriform) cortex onto two key structures involved in emotion and memory: the amygdala and hippocampus (Figure 1; Carmichael et al., 1994; Insausti et al., 1997; Haberly, 1998). In contrast with other sensory modalities, projections from the sensory input onto these two structures do not pass via the thalamus. From these areas, information is then conveyed to the secondary olfactory cortices composed of the orbitofrontal cortex (OFC) and the insular cortex.

The strong anatomical connection between olfactory and memory structures makes olfaction a privileged sense for accessing memories. However, in light of the amount of behavioral data describing the characteristics of episodic odor memory, the paucity of information available on the neural substrates of this function is startling. The purpose of this review is threefold: (1) to assess and discuss the current knowledge of the neural correlates of episodic odor memory by presenting functional data from healthy participants; (2) to describe the diversity of paradigms and therefore the diversity of cognitive processes by focusing on laboratory-based approaches, such as odor recognition memory and odor-associative memory, and on autobiographical approaches, such as the evaluation of odor familiarity and odor-evoked autobiographical memory; and (3) to point to new experimental and theoretical directions that episodic odor memory research could profitably pursue. To fulfill this triple objective, we choose to present the literature data according to experimental approaches and not to follow the chronological order of publications.

## Laboratory-based approaches for studying the neural bases of episodic odor memory

In laboratory-based approaches for studying episodic odor memory, participants artificially encounter odors in laboratory settings during a first phase (named the “encoding phase”), and then, the memory trace of this odor is questioned in a second phase (named the “test phase”). We will describe in detail three types of laboratory-based approaches to test episodic odor memory, with the level of complexity increasing from the memory of a single item (i.e., the odor recognition) to the memory of an odor using its verbal label (i.e., the odor-verbal recognition memory) and finally to the memory of an association between two items of different modalities (i.e., the crossmodal odor associative memory).

### Odor recognition memory

**Figure 2:**
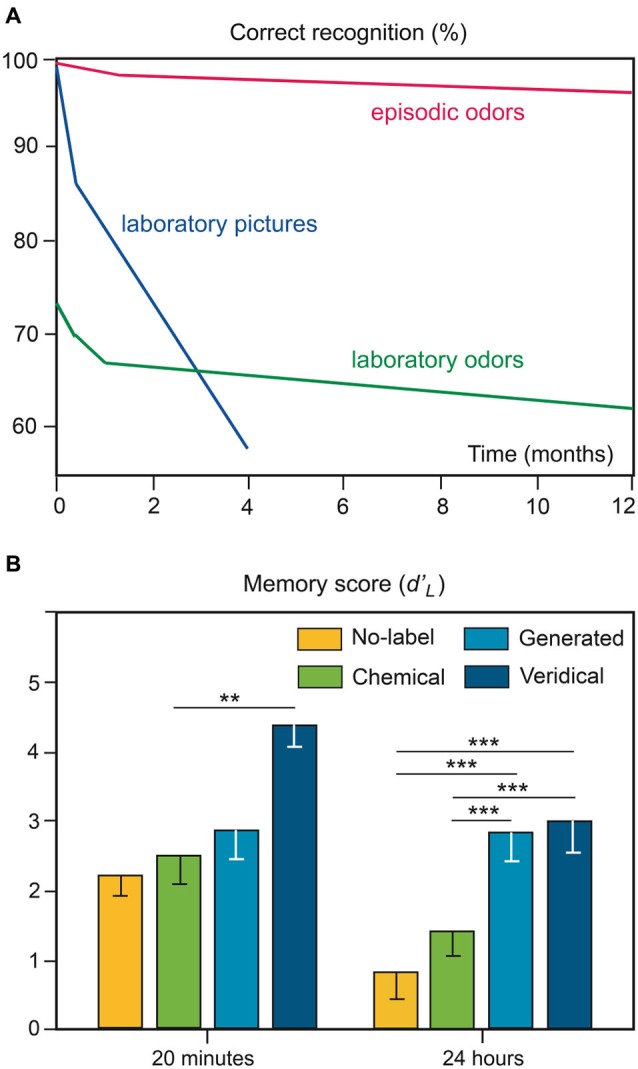
**Odor recognition memory. (A)** Ability to recognize laboratory pictures and odors over a span of 1 year. The hypothetical curve of the ability to recognize episodic odors (odors associated with significant real-life experiences) is shown for comparison (adapted from Engen, 1987). **(B)** Impact of semantic processing on odor recognition memory performances. Memory scores for odors that were previously associated with no labels, chemical labels, labels generated by the participants or veridical labels. ** *p* < 0.01; *** *p* < 0.001 (adapted from Jehl et al., 1997).

Recognition memory for odors received very little attention until the 1970s. The first study was led by Engen and Ross (1973). In this typical odor recognition paradigm, the participants were exposed to target odors in laboratory settings and, after a retention interval, were asked to decide whether the odor probe was an old stimulus (target odor) or a new one (distractor odor). This paradigm can be defined as investigating the ***explicit recognition of laboratory odors***. The authors demonstrated that the memory of odors has very little long-term loss. Laboratory odors were less well recognized than laboratory pictures after a short interval of time (73% correct recognition), but they were better recognized than these laboratory pictures after 4 months (Figure 2A; Engen, 1987). However, this specificity of odor recognition memory has been challenged more recently and significant forgetting of odors over time was observed (e.g., Murphy et al., 1991; Larsson, 1997; Olsson et al., 2009).

The robust ability to accurately recognize odors has been consistently demonstrated (e.g., Lawless and Cain, 1975; Lawless, 1978; Rabin and Cain, 1984; Goldman and Seamon, 1992). Nevertheless, as highlighted in Herz and Engen (1996), odor recognition performance strongly depends on the experimental conditions. First, the odor set size and odor similarities both affect odor recognition: a greater number of odors and a closer similarity among odors result in lower scores (Engen and Ross, 1973; Lawless and Cain, 1975; Jones et al., 1978; Schab, 1991). Second, the perceived qualities of odors influence recognition memory. For example, evidence suggests that the unpleasantness of odors and their high intensity improve the robustness of memories (Larsson et al., 2009). Third, performances in odor recognition are strongly and positively dependent on the amount of semantic information regarding the odor source, as observed in the influence of odor familiarity (Figure 2B) and odor-naming ability (e.g., Rabin and Cain, 1984; Lesschaeve and Issanchou, 1996; Jehl et al., 1997; Larsson and Backman, 1997; Bhalla et al., 2000; Frank et al., 2011). Fourth, recognition memory performances can also be affected by the type of procedure engaged in encoding. While no differences emerge for odors learned intentionally or incidentally (Engen and Ross, 1973; Larsson et al., 2003, 2006), the processing task used to encode odor affects the subsequent recognition of odors. Odors are better recognized after elaborative processing (verbal definition, association with a life episode) than after pure odor perceptual processing (Lyman and McDaniel, 1986, 1990). Thus, the importance of semantic processing in odor recognition must be taken into account and, as Schab (1991) previously noted, “*A more realistic assessment of the odor-recognition data reported in the literature, therefore, acknowledges that recognition performance is the joint result of memory for perceptual odor information and memory for covertly generated verbal associations to the odors*”.

Two states of awareness are thought to be involved in recognition memory retrieval: ***recollection***, which involves the remembering of an item along with contextual and associative details, and ***familiarity***, where an item is seen as familiar but no other contextual information is remembered (Mandler, 1980). The recollective experience is experimentally approached through the ***Remember/Know procedure*** (Tulving, 1985) in order to determine how much recollection and familiarity contribute to different kinds of recognition. The recollective experience occurring in odor recognition memory is influenced by several factors: odor familiarity and identifiability, and gender (Larsson et al., 2003, 2006; Olsson et al., 2009). For instance, Larsson et al. (2006) showed that recognition is more based on recollection than familiarity for familiar odors, and is more based on familiarity and guessing than on recollection for unfamiliar odors.

The neural basis of odor recognition memory has been approached in four studies using standard recognition memory tests. Two positron emission tomography (PET) studies, which were among the first neuroimaging studies on olfactory cognitive processes, highlighted the brain regions specifically involved in long-term odor recognition memory in comparison with short-term odor memory processes (Savic et al., 2000; Dade et al., 2002). These two studies noted the importance of the prefrontal and posterior-parietal regions in long-term odor memory. They also revealed the role of the PC, especially its right part, in odor recognition. This right-hemisphere superiority in odor recognition has also been reported in patients with brain lesions. Despite a few discrepancies (Hudry et al., 2003), either patients with right temporal lobe or right orbitofrontal lesions or those with right temporal lobe epilepsy perform more poorly than do patients with left-sided lesions in odor recognition tests (Rausch et al., 1977; Carroll et al., 1993; Jones-Gotman and Zatorre, 1993).

**Figure 3:**
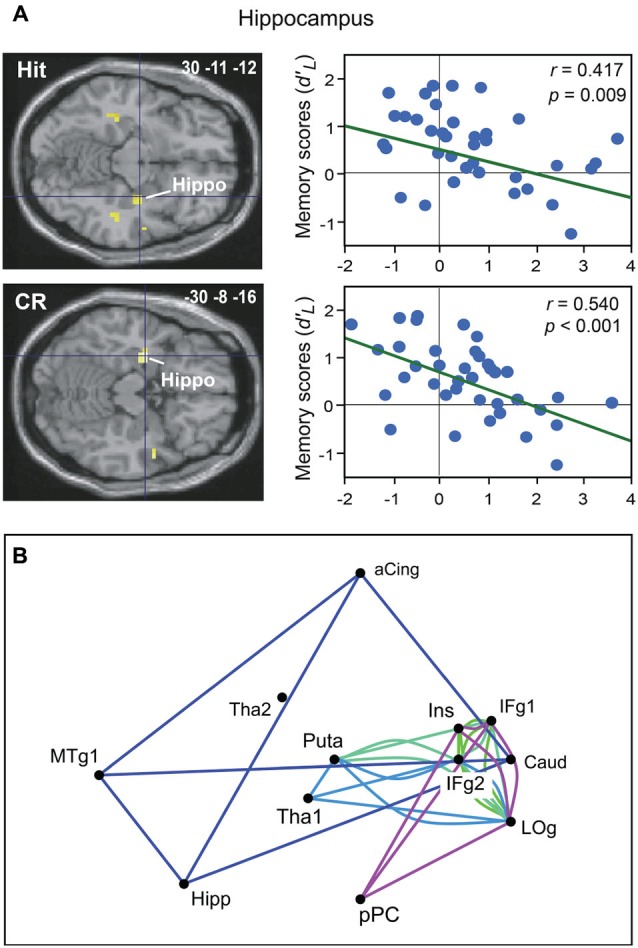
**Neural basis of odor recognition memory. (A)** Decreased activation intensity in the right and left hippocampus as a function of memory scores (*d’_L_*) for Hit and CR in all participants (adapted from Royet et al., 2011). **(B)** The module in dark blue shows four regions functionally connected during the Hit condition. Other modules were also found during the CR, Miss or False alarm conditions. aCing, anterior cingulate; Caud, caudate nucleus; Hipp, hippocampus; IFg, Inferior frontal gyrus; Ins, insula; LOg, lateral orbital gyrus; MTg, medial temporal gyrus; pPC, posterior piriform cortex; Puta, putamen; Tha, thalamus (adapted from Meunier et al., 2014).

Two of our studies recently further elucidated odor recognition memory by investigating the neural basis of this process as a function of task performance using event-related functional magnetic resonance imaging (fMRI; Royet et al., 2011; Meunier et al., 2014). Recognition memory performances were assessed using parameters from signal detection theory, which has widely dominated recognition memory theory since the 1950s (Swets, 1964; Lockhart and Murdock, 1970). From the experimental conditions (target *vs*. distractor) and the participants’ behavioral responses (“Yes” *vs*. “No”), four response categories were defined: Hit or Miss when the target items were accurately recognized or incorrectly rejected, respectively, and Correct Rejection (CR) or False Alarm when the distractor items were correctly rejected or incorrectly recognized, respectively. Using both standard and multivariate analyses, we observed that correct and incorrect recognition and rejection induced distinct neural signatures (Royet et al., 2011). Mainly, activity in the hippocampus and the parahippocampal gyrus was associated with the correct recognition of odors, whereas the perirhinal cortex was associated with errors in recognition and rejection. More strikingly, we observed a decreased involvement of the anterior hippocampus when memory performances increased during correct recognition and rejection (Figure 3A). These findings led to the hypothesis that a greater ease when performing the task results in less activation in the hippocampus. Recently, we explored the functional connectivity of the networks underpinning correct and incorrect olfactory memories using graph theory (Meunier et al., 2014). We found that among 36 regions of interest, the hippocampus, caudate nucleus, anterior cingulate and medial temporal gyrus were more frequently connected together during correct odor recognition and thus formed a specific module of this condition (Figure 3B). The poor odor recognition performances observed in patients with hippocampal lesions (Levy et al., 2004) agrees with the essential role of the hippocampus in odor recognition memory.

### Odor recognition memory from verbal labels

Odor recognition memory has also been investigated through the recognition of odor verbal labels where the odors are presented during the encoding phase and the odor labels are retrieval cues (Buchanan et al., 2003; Cerf-Ducastel and Murphy, 2006; Lehn et al., 2013). This paradigm can be defined as testing the ***explicit recognition of the verbal labels of laboratory odors*** and addresses the label-odor association. Although no statistical comparison was performed, the behavioral results depicted by Buchanan et al. (2003) suggested that the odor-verbal recognition paradigm leads to lower memory scores than those for the odor-odor recognition paradigm. This empirical observation indicates that odor recognition is more difficult when triggered by a label than by the odor itself.

**Figure 4:**
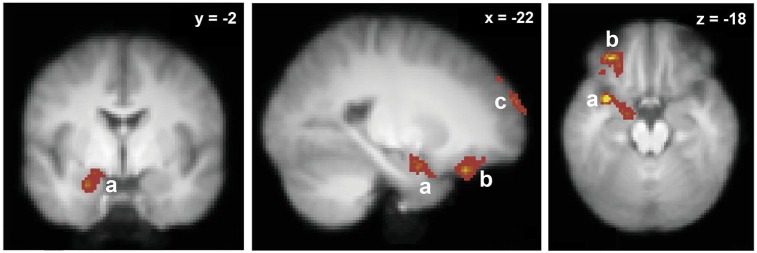
**Neural basis of odor recognition through verbal label**. Brain activations specific to olfactory modality (in comparison with object-verbal recognition). a, Left insula/amygdala/piriform cortex; b, left orbitofrontal cortex; c, left frontal pole (adapted from Lehn et al., 2013).

The neural substrates of odor retrieval through odor name recognition have been investigated a couple of times (Cerf-Ducastel and Murphy, 2006; Lehn et al., 2013). The two studies were consistent with regards to the ensemble of brain regions involved in this odor memory process and revealed consistent activation in the hippocampus, PC, amygdala, OFC and cerebellum. However, comparing odor-name and object-name recognition memories, Lehn et al. (2013) further showed that the hippocampus was activated during the recognition memory of both types of cues, thus providing clear evidence for modality-independent functions of the hippocampus. In turn, a region encompassing the left anterior insula, PC and amygdala, in addition to the left OFC, the left frontal pole and the right cerebellum, were specific to the olfactory modality (Figure 4).

An advantage of using verbal cues is the facilitation of crossmodal comparisons because identical sensory inputs (retrieval cues) are used for different types of stimuli (Lehn et al., 2013). However, the main drawback of this technique is the typically weak link between an odor and its verbal label (Lawless and Cain, 1975; Engen, 1987). Humans perform poorly when identifying common odors from smell alone (Engen and Pfaffmann, 1960; Cain, 1979). This difficulty makes the recognition more complex. When a verbal label is presented during the retrieval phase, two strategies can be implemented. The participants can compare the label they were reading to all the labels explicitly or implicitly generated during the encoding phase, a task that involves semantic-based recognition memory. They can also decide whether the odor evoked by the test label matches the memory trace of the encoded odors, a task that refers to an episodic-based recognition memory. Thus, the use of a verbal label to test odor recognition obscures the nature of the memory processes involved during retrieval.

### Crossmodal odor associative memory

In contrast to odor recognition memory from the odor label, crossmodal odor associative memory is related to the association of an odor with a non-odor item. The capacity of healthy adult volunteers to retrieve associations between two items, including an odor, has been demonstrated through two main paradigms. The ***paired-associate paradigm*** tests the ability to recall the item previously associated with an odor during explicit encoding. Davis (1975, 1977) showed a disadvantage for odors as associative stimuli in comparison with abstract visual stimuli. However, they also observed that this disadvantage decreased with higher odor familiarity and with higher dissimilarity within odor sets, a result that is consistent with the observations reported above in terms of the impact of familiarity and qualitative similarity on odor recognition memory performances (see Section Odor Recognition Memory). The ***odor source paradigm*** tests the ability to retrieve limited contextual information associated with the odor perception during encoding. For instance, participants were asked to explicitly remember either a specific room (Takahashi, 2003) or a specific space on a board (Gilbert et al., 2008; Pirogovsky et al., 2009) in which the odors were initially presented or to remember the gender of the experimenter presenting the odors during the encoding phase (Gilbert et al., 2006; Pirogovsky et al., 2006; Hernandez et al., 2008). Overall, these studies demonstrated that odor recognition is superior to the recognition of the source, that explicit *vs.* implicit encoding improves the memory for the source but not for the odor itself, and that aging affects odor source memory than on odor recognition (Takahashi, 2003; Gilbert et al., 2006, 2008; Pirogovsky et al., 2006, 2009; Hernandez et al., 2008).

Functionally, crossmodal odor associative memory has been investigated only twice using the paired-associate paradigm. In the study led by Gottfried et al. (2004), objects were paired with odors, and the participants were instructed to imagine a link between each object and the smell (*a priori*, the objects had no explicit link with odor). The effect of “odor context” on the neural responses was then examined during retrieval when these same objects were presented among distractors. In other words, this paradigm studied the implicit recall of the odor through the explicit recognition of the object that was previously paired with the odor but not the conscious retrieval of the odor. This memory process can be defined as an ***implicit crossmodal recall of laboratory odor context***. Gottfried et al. (2004) showed evidence for the reactivation of the right posterior PC during successful object recognition in the absence of olfactory stimulation, just by the specific reactivation of the association between the recognized object and its paired odor. The authors further demonstrated that the involvement of the primary olfactory cortex is independent of the odor valence and that this structure is more sensitive to the retrieval of odor than the retrieval of visual stimuli. More importantly, the authors found that odor retrieval involved the right anterior hippocampus, and hence hypothesized that this structure has an important role in the binding between both items. A recent neuropsychology study supports this hypothesis and shows that amnesic subjects with hippocampal damage have impaired odor-place memory but intact odor recognition (Goodrich-Hunsaker et al., 2009). Yeshurun et al. (2009) also suggested a specific role of the hippocampus for odor associative memory. They based their study on the finding that the first odor-to-object association is stronger than subsequent associations of the same odor with other objects (Lawless and Engen, 1977). They paired object photos twice with a different odor, a different sound or a different odor-sound stimulus each time. One week later, the participants were presented with the object photos and had to explicitly recognize, among distractors, the odor associated with the object during encoding through odor labels. This task can be defined as investigating the ***explicit crossmodal recognition of laboratory odor context***. Yeshurun et al. (2009) observed hippocampal activation for early olfactory but not auditory associations regardless of whether they were pleasant or unpleasant. These findings confirmed the hypothesis that the first olfactory associations enjoy a privileged brain representation that is underlined by the hippocampus.

The odor associative memory paradigms allow the examination of long-term odor memory involving more complex processes than those implicated in the memory of a single item (i.e., odor recognition memory). In these paradigms, the memory concerns the association between an item and a given context. However, the richness of the context is usually limited and materialized by a single other dimension. Therefore, the gap between odor associative memory and odor autobiographical memory is still wide. As highlighted by Schab (1991) “*the conditions under which an odor often is reported to evoke the recollection of past episode differ significantly from those of a paired-associate task. In the former, a single ambient odor triggers the remembrance of a personal episode of which the odor itself was an integral part, whereas in the latter a series of different odors is presented, typically in small bottles, and the learning task is deliberate and requires the acquisition of unrelated and personally irrelevant information*”.

## Autobiographical approaches for studying the neural basis of episodic odor memory

In odor-evoked autobiographical approaches, the content of the memory refers to the participants’ past, and its retrieval is triggered with odors. First, we will present the experiments that questioned the memory of previously encountered odors and investigated the feeling of familiarity and unfamiliarity. Then, we will present the studies that addressed the recall of real-life events and investigated odor-evoked autobiographical memories.

### Feeling of familiarity of odors

Odor autobiographical memory can be investigated through the feeling of familiarity generated by odors that are presented in laboratory settings. This paradigm refers to the ***explicit recognition of self-relevant odor***. As we previously described, “*The feeling of familiarity is a long-term recognition memory process referring to a subjective state of awareness based on judgments of the item’s prior occurrence. It involves the recognition of the item’s perceptual features and eventually of conceptual or semantic features, without the confirmatory conscious recollection of contextual information and/or without identification*” (Plailly et al., 2007). A consensus emerges from the evaluation of odor perceptual characteristics. There is consistent evidence for positive correlations between the ratings of odor familiarity and those of intensity and pleasantness (e.g., Jellinek and Köster, 1983; Ayabe-Kanamura et al., 1998; Distel et al., 1999; Royet et al., 1999). Familiar odors have also been described as more simple, in terms of ease of interpreting an odor meaningfully (Sulmont et al., 2002). Recently, Delplanque et al. (2008) argued that the relation between pleasantness and familiarity is nonlinear: pleasantness ratings were positively correlated with familiarity ratings for pleasant odors, but not for unpleasant odors, a result that has been subsequently replicated (Plailly et al., 2011; Ferdenzi et al., 2013).

Our research team was the first to address the neural basis of the familiarity process. In the first studies, we compared periods of brain activity recorded when participants rated the familiarity of a large set of familiar or unfamiliar odors to periods when they detected the presence of odors (Royet et al., 1999, 2001; Plailly et al., 2005). Participants were instructed to make familiarity judgments based on their life experiences (i.e., “*Does this odor seem familiar to you?”*). This paradigm avoided the need for an initial experimental encoding phase. Greater activation of the right OFC and the right PC was observed when the participants evaluated odor familiarity compared with when they detected odors (Royet et al., 1999, 2011; Plailly et al., 2005). The lateralization of this memory process (Royet and Plailly, 2004) was consistent with the higher familiarity of odors presented to the right nostril than those presented to the left nostril (Broman et al., 2001). This could also explain the right hemisphere lateralization of the odor process observed in the first studies when odorants were passively perceived because the odorants were familiar and could have automatically triggered recognition (e.g., Zatorre et al., 1992; Yousem et al., 1997; Sobel et al., 1998; Savic et al., 2000; Poellinger et al., 2001). Our studies on odor familiarity evaluation further emphasized the role of the left inferior frontal gyrus, a key region for semantic processing, which is most likely activated in an attempt to gather semantic information to identify the smell (Royet et al., 1999, 2011; Plailly et al., 2005). Additional activations were observed in the brain regions involved in emotion (amygdala), visual mental imagery (fusiform and occipital gyri) and memory (hippocampus and parahippocampal gyrus) processes, reflecting the large set of cognitive processes engaged during the evaluation of odor familiarity (Plailly et al., 2005).

**Figure 5:**
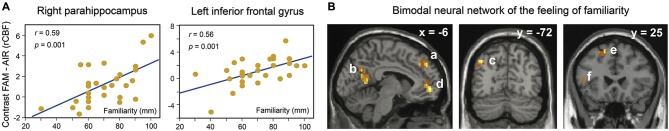
**Neural basis of odor familiarity. (A)** Correlations between familiarity ratings and activation in the right parahippocampus and left inferior frontal gyrus. The y-axis denotes differences in regional cerebral blood flow (rCBF) between the familiarity and baseline conditions (FAM–AIR). The x-axis shows the mean familiarity ratings of four familiar and four unfamiliar odorants for each participant (adapted from Savic and Berglund, 2004). **(B)** Bimodal neural basis of the feeling of familiarity evoked by odor and music (in comparison with the feeling of unfamiliarity). a, superior frontal gyrus; b, precuneus; c, angular gyrus; d, superior frontal gyrus bordering the cingulate gyrus; e, superior/middle frontal gyrus; f, inferior frontal gyrus. All regions were in the left hemisphere. The hippocampus and parahippocampal gyrus were regions of interests and hence were not displayed (adapted from Plailly et al., 2007).

Savic and Berglund (2004) and Plailly et al. (2007) revealed that familiar and unfamiliar odors are processed by different neural circuits. Savic and Berglund (2004) reported that the passive perception of odorants selected to be familiar *vs.* unfamiliar elicited specific activation of the right parahippocampal gyrus, right middle and inferior temporal gyri, and the left parietal cortex covering the precuneus. In addition, the familiarity ratings obtained after functional acquisitions were positively correlated with activation in the left inferior frontal gyrus and the right parahippocampal gyrus (Figure 5A), suggesting that the smelling of familiar, but not that of unfamiliar, odors engages neural circuits mediating semantic association and episodic retrieval functions. Our research team completed the preceding results by unveiling the existence of a bimodal neural system engaged in the feeling of familiarity *vs.* unfamiliarity (Plailly et al., 2007). The neural correlates of self-rated familiarity evoked by items of two modalities, odors and musical excerpts, overlapped within an extensive bimodal neural system that included the prefrontal, inferior frontal, parieto-occipital and medial temporal lobe brain regions in the left hemisphere (Figure 5B). We further concluded that because this system also overlaps with the familiarity processing of other types of stimuli (i.e., faces, voices, pictures and verbal items), a multimodal neural network might underlie the feeling of familiarity. Interestingly, we revealed the existence of neural processes specific to the feeling of unfamiliarity, which might be related to the detection of novelty, with a main bimodal activation in the right insula.

### Odor-evoked autobiographical memory

Odor-evoked autobiographical memory can be investigated through the recall of the life episode associated with an odor. This paradigm refers to the ***explicit recall of autobiographical memories evoked by self-relevant odor***. Odors are exceptional cues for evoking personal autobiographical memories. Behavioral evidence has demonstrated that odors are more effective triggers of emotional memories than the same cue presented in other sensory formats or even in the form of odor labels (Hinton and Henley, 1993; Chu and Downes, 2002; Herz and Schooler, 2002; Herz, 2004, 2012; Herz et al., 2004; Larsson and Willander, 2009; Arshamian et al., 2013). Another specificity of odor-evoked autobiographical memories is that they produce a unique age distribution and favor childhood memories stemming from the first decade of life rather than from young adulthood, which is the typical reminiscence bump for memories evoked by verbal and visual information (Chu and Downes, 2000; Willander and Larsson, 2006; Larsson and Willander, 2009; Miles and Berntsen, 2011). Furthermore, empirical evidence indicates that odor-evoked memories are associated with stronger feelings of being brought back in time (Herz and Schooler, 2002; Herz, 2004; Willander and Larsson, 2006, 2007; Arshamian et al., 2013) and are thought of and talked about less than memories elicited by visual or verbal variants of the same items (Rubin et al., 1984). Finally, odors may also be more likely than visual or verbal cues to elicit perceptual-based memories; visual or verbal cues in turn provide more conceptual-based memories (Herz and Cupchik, 1992; Goddard et al., 2005; Willander and Larsson, 2007).

**Figure 6:**
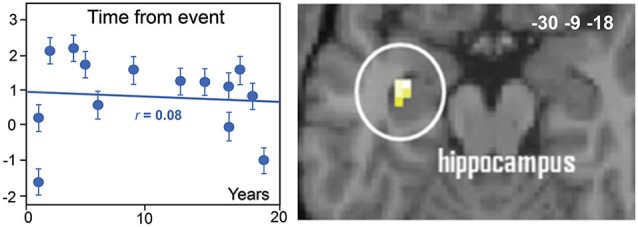
**Neural basis of odor-evoked autobiographical memory**. The blood oxygen level-dependent signal in the hippocampus region of interest did not significantly vary with the time from the event (adapted from Arshamian et al., 2013).

Although the high potential of odors to generate the successful recall of autobiographical memories has been behaviorally demonstrated, the neural basis remains little explored. Only two studies have investigated the neural underpinnings of odor-evoked autobiographical memories. Herz et al. (2004) explored whether the brain correlates of personal memories elicited by the smell of a perfume were different from those elicited by the sight of this perfume. Arshamian et al. (2013) compared memories evoked by either personally meaningful odors or pleasant control odors. In both studies, the authors observed activation in the parahippocampal gyrus, the amygdala, and the middle occipital gyrus. These regions play a crucial role in memory, emotion and visual mental imagery, and their engagement could explain the fact that odors are especially potent reminders of autobiographical experiences. Interestingly, Arshamian et al. (2013) raised two important issues. The first was inspired by the debate opposing ***the multiple memory trace theory consolidation model*** that postulates that the hippocampus and neocortex are in constant interaction (Nadel and Moscovitch, 1997, 1998) and the ***standard model of memory consolidation*** where the passage of time leads to a disengagement of the hippocampus and an additional recruitment of the prefrontal cortex (Marr, 1971; Squire et al., 1984). Arshamian et al. (2013) observed that hippocampal activation did not vary as a function of memory remoteness, which supports the notion of a permanent role of the hippocampus in the retrieval of odor-evoked autobiographical memories (Figure 6). Second, because of the early reminiscence bump in olfaction, the authors tested whether odors were differentially coded depending on the decade in which the stimulus was encoded. They observed a greater involvement of regions devoted to perceptual processes (e.g., the orbitofrontal cortex) during the recall of first-decade odor-evoked memories and a greater recruitment of regions involved in semantic processing (the left inferior frontal gyrus) during the recall of second-decade odor-evoked memories. This result suggests that the autobiographical recall is based more on perceptual processing and less on semantic processing when memories refer to early life experiences.

## Laboratory-ecological approaches for studying the neural basis of episodic odor memory

The two main approaches for studying episodic memory developed above, the laboratory-based and autobiographical approaches, each have pros and cons. In the laboratory-based approach, artificial and simple episodes are encoded and recalled in controlled conditions in the laboratory. This method enables the manipulation of the encoding conditions and the retention time and allows the oversight of recall veracity. However, the to-be-remembered materials that are developed by experimenters are poor in comparison with a real-life episode. In the autobiographical approach, the retrieval of real-life memories that are encoded in the participants’ past is triggered by an experimental cue. This approach allows for the recall of real-life events in quite ecological conditions, but the veracity of the recalled events cannot be controlled. McDermott et al. (2009) have underscored the interest in proposing a new approach to study and understand human episodic memory, one that is halfway between the two traditional approaches and retains the respective advantages of each. Fulfilling those expectations, several ***laboratory-ecological approaches*** have been recently devised to study episodic memory (Pause et al., 2010, 2013; Holland and Smulders, 2011; Milton et al., 2011; Easton et al., 2012; Saive et al., 2013). On the one hand, these approaches are close to Tulving’s definition of episodic memory (Tulving, 1972, 1983) by allowing the conscious and controlled recollection of specific and complex events from the past. On the other hand, they are derived from content-based approaches developed in animals proposing to define the content of episodic memory as ***What happened***, ***Where*** and ***When*** (Clayton and Dickinson, 1998; Griffiths and Clayton, 2001; Babb and Crystal, 2006; Crystal, 2009). In addition to the three-dimensional content of the episodic memories, Clayton et al. (2003) argued that these memories must also be integrated, flexible and trial unique. Subsequently, Easton and Eacott (2008; Eacott and Easton, 2010) enriched this operational definition of episodic memory by considering an alternative to the temporal dimension. They proposed replacing this dimension by the specific occasion or context in which the event occurred (***Which context***); this context encompasses the time when important but also the emotion, semantic knowledge, visual scene, or auditory and olfactory environments.

In the study of episodic odor memory, the laboratory-ecological approaches are still rare, although the necessity to elaborate new paradigms has been raised for more than 20 years. Schab (1991) wrote that “*discrepancy between experience and past experimental research is due to less than optimal choice of procedures in the laboratory studies. One means of studying odor-cued recall in the laboratory is to ‘create’ a personal significant event*”. This insight led Schab and Cain (1992) to suggest an example of a laboratory-based, personally significant event, which consisted of a scenario during which the participants witness a specific emotional event in the context of ambient odor and sound. This emotional event could be tested later to investigate the power of odor *vs.* sound to evoke episodic memory retrieval. The authors hypothesized that “*Such an experiment might support the popular expectation regarding odor-evoked retrieval because it may stimulate the environmentally realistic event more faithfully*”. However, their reflections did not give rise to any experiment. Sometime later, Aggleton and Waskett (1999) imagined an ingenious experiment where visitors to a museum were re-exposed to the ambient smell of a previous exhibition and were questioned about their memories of this exhibition. The odor specifically acted as an effective retrieval cue and improved their memory performances. This approach allowed for the investigation of the retrieval of a real-world episode but not in its entirety. The authors only tested the content of the exhibition and not the context or the emotion associated with the event. Along the same lines, Herz and Cupchik (1995) and Herz (1998) attempted to address the power of emotion triggered by odor to induce the recall of a memories-like association created in the laboratory. They used a paired-associate paradigm in which emotional paintings or pictures were paired with emotional odors or a verbal, visual, musical or tactile variant of the same cue. The mean percentages of paintings or pictures correctly recalled were similar across modalities, but the odor-evoked memories were significantly more emotionally loaded than the memories cued by the other modalities. The directions toward which this experiment went were exciting, but they were not further developed. Additionally, the paradigm was never enriched to match the content-based episodic-like memory definitions (Tulving, 1972; Easton and Eacott, 2008).

**Figure 7:**
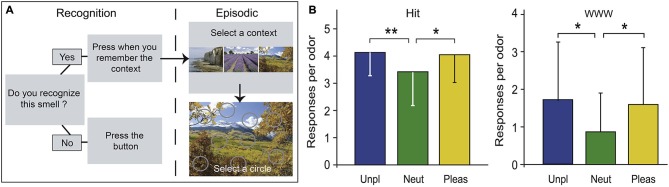
**Laboratory-ecological approach for studying episodic memory. (A)** Episodic-memory task design. The memory of the episodes was tested using an odor recognition task followed for the “Yes” trials by an episodic memory retrieval (selection of a visual context and a location). **(B)** Number of accurate odor recognitions (Hit) and accurate episodic retrievals (WWW) as a function of odor pleasantness. Neut, neutral; Pleas, pleasant; Unp, unpleasant. * *p* < 0.05; ** *p* < 0.01 (adapted from Saive et al., 2014).

To investigate odor-evoked episodic memory, we recently developed an original laboratory-ecological approach deeply inspired by episodic-like memory tasks performed by animals (Saive et al., 2013). It was as ecologically valid as possible, yet the encoding and retrieval conditions were fully controlled. The to-be-remembered episodes were trial-unique, rich, close to real-life episodes, and in agreement with the definitions of episodic memory proposed by Tulving (1972) and Easton and Eacott (2008). During the encoding phase, the participants freely explored three unique episodes, one episode per day. Each unique episode was composed of three unfamiliar odors (What) positioned at three specific locations (Where) within a visual context (i.e., a picture of a landscape; Which context). We intentionally selected unfamiliar and largely unidentifiable odors and arbitrarily linked the odors, spatial locations and visual contexts in each episode to limit associative semantic processes. On the fourth day, the odors were used to trigger the retrieval of the complex episodes in a recall test. The participants were asked to recognize odors and to correctly remember the visuospatial context in which they were encountered, ensuring the evaluation of the memory content accuracy (Figure 7A). The participants were highly proficient in recognizing the target odors among distractors and retrieving the spatio-contextual environment of the episode with a rather high confidence level (Saive et al., 2013). This observation suggests that when an association between odors, spatial locations and contexts is encoded, the association forms an integrated representation retrievable by the participants. More recently, using a similar procedure, we observed that memory performances were influenced by the emotional content of the odor, regardless of their valence; both pleasant and unpleasant odors generated greater recognition and episodic retrieval than did neutral odors (Figure 7B; Saive et al., 2014). Our new approach is adapted to fMRI constraints and should permit further investigations of the neural basis of episodic odor memory.

## Conclusion and future directions

Episodic odor memory is experimentally studied through a large set of paradigms and, as a consequence, the concept of “episodic odor memory” refers to an ensemble of memory processes which varied in complexity from the recognition of a single odor to the autobiographical memory evoked by odor. While large conceptual differences distinguish the laboratory-based and the autobiographical approaches, each approach has specificities that are complementary to the understanding of the neural underpinnings of the episodic odor memory. In laboratory-based approaches, the content of the memory is fully controlled and brain signals can be analyzed regarding the accuracy of the participants’ responses, allowing for the distinction between the neural substrates related to memory success or to memory failures. For example, a module of tightly-connected brain regions (hippocampus, caudate nucleus, anterior cingulate and medial temporal gyrus) is specifically involved when odors are accurately recognized (Meunier et al., 2014), while the perirhinal cortex is specifically associated with memory errors (Royet et al., 2011). In autobiographical approaches, the access to real-life memories allows for the involvement of a wider ensemble of cognitive processes. The personal significance of the cue item generates the engagement of semantic processes, as highlighted by the role of the inferior frontal gyrus (Royet et al., 1999, 2011; Savic and Berglund, 2004; Plailly et al., 2005, 2007), and of emotional and visual imagery processes reflecting the vividness of the recalled memories (Herz et al., 2004; Plailly et al., 2005). Studying autobiographical memories also enables addressing consolidation process over time and suggests a continuous engagement of the hippocampus whatever the age of the memory (Arshamian et al., 2013).

While the two experimental approaches differ in their conception of episodic memory, the overview of the functional neuroimaging findings suggests a core of relatively stable neural correlates of episodic odor memory regardless of the approach. The major role of the PC in human episodic odor memory is consensual. This finding agrees with the associational properties of the primary olfactory cortex observed in animals (Litaudon et al., 1997; Haberly, 2001; Wilson and Stevenson, 2003) and its role in working odor memory in humans (Zelano et al., 2009). The involvement of the PC in episodic odor memory is modality-specific (Gottfried et al., 2004; Lehn et al., 2013), it is independent of odor valence (Gottfried et al., 2004; Yeshurun et al., 2009), and it tends to be lateralized to the right (vs. left) hemisphere (Savic et al., 2000; Dade et al., 2002; Gottfried et al., 2004; Plailly et al., 2005; Cerf-Ducastel and Murphy, 2006). The hippocampus is also consistently observed in both approaches, which is consistent with a large amount of literature that stresses the importance of this brain region in episodic memory (e.g., Suzuki and Amaral, 1994; Burwell and Amaral, 1998; Witter et al., 2000; Squire et al., 2004; Davachi, 2006; Diana et al., 2007; Eichenbaum et al., 2007). The literature involving the olfactory modality further shows that hippocampal activation reflects the memory performance (Royet et al., 2011; Lehn et al., 2013), and that while the hippocampus is engaged in the episodic memory of different sensory modalities (Plailly et al., 2007; Lehn et al., 2013), it has a privileged role for the first olfactory associations (Yeshurun et al., 2009). Additionally to the PC and hippocampus, laboratory-based and autobiographical approaches are concordant in the role of prefrontal, infero-temporal, postero-parietal and medial temporal lobe brain regions in odor episodic memory. Thus, the present review agrees with previous report demonstrating that brain networks involved in classical autobiographical studies partially overlap with those found in more controlled laboratory episodic memory tasks (Cabeza et al., 2004; Burianova and Grady, 2007; Cabeza and St Jacques, 2007).

We believe that the development of laboratory-ecological approaches that control the encoding and retrieval of specific and multidimensional laboratory episodes can yield new discoveries for the comprehension of episodic memory. By controlling each aspect of the to-be-remembered event and of its retrieval, specific questions can be addressed. For example, the close relationship between olfaction, emotion and memory, commonly illustrated as the Proust phenomenon (Chu and Downes, 2000), can be further explored by manipulating the emotional strength of the episode during encoding and by manipulating the sensory modality of the cue that triggers episodic retrieval during the test phase. Furthermore, Mitchell and Johnson (2009) stressed the importance to rate amount of details of various types or vividness, emotional valence, arousal, because they provide specific information that explain the complex inter-play of cognitive processes that are characteristic when retrieving rich memories and that can be related to brain activity. Such features are relatively easy to measure and can be crucial in the understanding of the different processes underlying episodic memory. We further suggest the investigation of the brain as whole through the use of specific analysis techniques. Most cerebral imaging functional studies have used univariate statistical analyses to localize individual aspects of brain function, and have restricted investigation to specialized cognitive sub-systems. Various techniques for measuring functional connectivity are to date available and their use can represent a considerable improvement in the understanding of episodic memory. This sum of efforts will be the basis of real advances in this field and will bring substantial progress in the understanding of the behavioral specificities of episodic odor memory.

## Conflict of interest statement

The authors declare that the research was conducted in the absence of any commercial or financial relationships that could be construed as a potential conflict of interest.

## Abbreviations

fMRI: functional magnetic resonance imaging
PET: positron emission tomography.
